# Psychosocial working conditions and the risk of diagnosed depression: a Swedish register-based study

**DOI:** 10.1017/S003329172100060X

**Published:** 2022-11

**Authors:** Melody Almroth, Tomas Hemmingsson, Alma Sörberg Wallin, Katarina Kjellberg, Bo Burström, Daniel Falkstedt

**Affiliations:** 1 Institute of Environmental Medicine, Karolinska Institutet, Stockholm, Sweden; 2 Centre for Social Research on Alcohol and Drugs, Stockholm University, Stockholm, Sweden; 3 Department of Global Public health, Karolinska Institutet, Stockholm, Sweden; 4 Centre for Occupational and Environmental Medicine, Region Stockholm, Stockholm, Sweden

**Keywords:** Depression, job control, job demands, job strain, occupational health

## Abstract

**Background:**

High job demands, low job control, and their combination (job strain) may increase workers' risk of depression. Previous research is limited by small populations, not controlling for previous depression, and relying on the same informant for reporting exposure and outcome. This study aims to examine the relationship between objectively measured workplace factors and the risk of developing clinical depression among the Swedish working population while controlling for previous psychiatric diagnoses and sociodemographic factors.

**Methods:**

Control, demands, and job strain were measured using the Swedish Job Exposure Matrix (JEM) measuring psychosocial workload linked to around 3 million individuals based on their occupational titles in 2005. Cox regression models were built to estimate associations between these factors and diagnoses of depression recorded in patient registers.

**Results:**

Lower job control was associated with an increased risk of developing depression (HR 1.43, 95% CI 1.39–1.48 and HR 1.27, 95% CI 1.24–1.30 for men and women with the lowest control, respectively), and this showed a dose–response relationship among men. Having high job demands was associated with a slight decrease in depression risk for men and women. High strain and passive jobs (both low control jobs) were associated with an increased risk of depression among men, and passive jobs were associated with an increased risk among women.

**Conclusion:**

High job control appears important for reducing the risk of developing depression even when accounting for previous psychiatric diagnoses and sociodemographic factors. This is an important finding concerning strategies to improve occupational and in turn mental health.

## Introduction

Depressive disorders are one of the leading causes of morbidity globally [Global Burden of Disease (GBD) 2017 Disease and Injury Incidence and Prevalence Collaborators, 2018; World Health Organization, 2017]. This pattern is clear in Europe (Andlin-Sobocki, Jonsson, Wittchen, & Olesen, 2005), and in Sweden, the incidence and indirect costs of depressive disorders have increased over the last decades (Tiainen & Rehnberg, 2009). Depression represents not only a large cost to individuals, but also the people around them and society as a whole.

For this reason, a better understanding of potentially modifiable risk factors is crucial in order to devise strategies for the prevention and improvement of depression. One such factor is the working environment, where most adults spend much of their time. Psychosocial characteristics of the work environment may be important for the development of depressive symptoms, as has been concluded in several systematic reviews (Bonde, 2008; Madsen et al., 2017; Netterstrom et al., 2008; Siegrist, 2008; Theorell et al., 2015).

One of the most important and widely used theoretical models for understanding psychosocial working conditions in relation to stress and health is the job demand–control or job strain model (Karasek, 1979). Job demands refer to the amount of work and time pressure that is experienced, while job control refers to how much influence a person has over the organization and pace of their work. Jobs that have high demands in combination with low control are known as high strain jobs, and this has been found to be associated with adverse health outcomes (Kivimäki et al., 2012).

While previous systematic reviews and meta-analyses have concluded that poor work environments regarding these psychological components are related to an increased risk of depression (Bonde, 2008; Madsen et al., 2017; Netterstrom et al., 2008; Siegrist, 2008; Theorell et al., 2015), the studies included in these reviews have some important methodological limitations. These limitations include inadequate control for confounding, relying on self-report of workplace exposures and/or depression and sometimes using the same informant for measuring both, and small sample sizes or sampling limited to a single branch or firm. These reviews conclude the need for independent measures of exposure and outcome and more objective measures of depression such as clinical diagnosis. Controlling for confounding is particularly important because there are several factors that may influence entrance and position in the labor market and risk for developing depression. Some such factors are disadvantages during childhood, immigrant status, education, family situation, and previous psychiatric health. One approach to independently measuring the psychosocial workplace conditions is the use of a Job Exposure Matrix (JEM). This approach surveys individuals within different occupations and aggregates their answers about exposures on an occupational level. These JEM scores can then be connected to individuals based on their occupations and have the potential to be used in population-level studies using more objective diagnostic healthcare data.

Some experts have argued that job control may be the more important factor in the job strain model in predicting health outcomes (Ingre, 2015; Knardahl et al., 2017; Mikkelsen et al., 2018), and that job control may actually be the reason for observing significant associations for the combined job strain variable. This, however, is not agreed upon by all (Kivimäki, Nyberg, & Kawachi, 2015). Possibly for this reason, some researchers choose to focus only on job control (Svane-Petersen et al., 2020). The different aspects of the model and their combination are most often expressed as a single dichotomous measure, which can make interpretation difficult. It is important to closely investigate a more extensive range of the different components of this model as well as their combination in order to better understand the relationship between job strain and depression and investigate possible dose–response relationships.

The present study aims to investigate the relationship between the psychosocial work environment exposures of job control, job demands, and job strain and the risk of developing depression during a 10-year follow-up period using aggregated job exposure data and patient registry information on the Swedish working population age 30–60 in 2005, taking into account previous psychiatric diagnoses, family situation, birth country, socioeconomic position (SEP) during childhood, and parents' mental health. Because of Sweden's relatively gender-segregated labor market (Hansen & Wahlberg, 2008) and important differences in potential workplace exposures, associations are considered separately for men and women.

Based on Karasek's job strain model (1979), we hypothesize that low job control and high job demands will independently relate to an increased risk of depression in a dose–response pattern, and that their combination (job strain) will also be associated with an increased risk of depression. We expect these associations to be partially explained by the above-mentioned background factors and for the pattern to be similar for men and women, though the strength of associations may vary.

## Methods

### Study population

The present study is based on the linkage between the Swedish total population register, the Longitudinal Integrated Database for Health Insurance and Labor Market Studies register (LISA), the Swedish National Patient Register, as well as census information from 1960, 1970, and 1980.

The total population register includes information on births, deaths, civil status, and migration and the LISA register includes mandatory reported information on occupation for everyone 16 years of age or older as well as their obtained education (Ludvigsson, Svedberg, Olen, Bruze, & Neovius, 2019). The national in-patient register has existed since the 1960s and has included psychiatric hospitalizations since 1973, while the out-patient register includes less severe public and private psychiatric visits and has been in use since 2001 (Socialstyrelsen, 2019). These Swedish registers have been commended for their accuracy and completeness in reporting (Ludvigsson et al., 2011, 2016, 2019).

The initial cohort resulting from these linkages, which has recently been named the Swedish Work, Illness, and labor-market Participation (SWIP) cohort, consists of around 5.4 million individuals. The present study is restricted to those born between 1945 and 1975 (i.e. between the ages of 30 and 60 at the baseline year, 2005). This restriction was made because it is assumed that those in this age group would have been both established and remaining in the labor market. We assume that those over the age of 30 are more likely to be stable in their occupations during the follow-up period compared to those under 30. The resulting population consists of around 3.8 million individuals of which around 3 million had complete information on occupation in 2005.

Ethical approval was obtained from the Stockholm ethics review board, reference number 2017/1224-31 and 2018/1675-32.

### Measures

#### Exposures

*Job control* and *job demands* were measured using the Swedish JEM measuring psychosocial workload based on information from the Swedish Work Environment Surveys (1997–2013). This JEM measures the aggregated experience of aspects of the work environment in different occupations separately for men and women. These scores are based on around 75 000 respondents and linked to a person's occupation based on the Swedish ISCO-88 four-digit classification of occupations obtained from the LISA register in 2005 (SCB statistics Sweden, 2001). Job control is measured based on four questions measuring decision authority, which is related to the amount of influence people have over the way their work is done. These questions focus on the aspects of the ability to determine which tasks to do, the pace of work, when to take breaks, and the structure of the work. Job demands are measured using three questions focused on the stress, time, and level of concentration of the job. The translated items are shown in Table 1, and extensive information on the construction of these JEMs is described in detail elsewhere (Fredlund, Hallqvist, & Diderichsen, 2000). These measures were initially scored as a mean for each occupation. We categorized these mean scores according to their quintile distribution separately for men and women, resulting in five categories ranging from low to high.

**Table 1.** Translated items used for the decision authority and demands Job Exposure Matrices (JEM)

JEM	Question	Answer alternatives
Decision authority	Can you partially decide when tasks should be done?	Never, mostly not, mostly, always
	Do you have the opportunity to decide your own work pace?	Not at all, occasionally, roughly ¼ of the time, half the time, roughly ¾ of the time, almost all the time
	Can you take short breaks to talk pretty much any time?	Not at all, occasionally, roughly ¼ of the time, half the time, roughly ¾ of the time, almost all the time
	Are you ever involved in deciding how your work is organzed?	Never, mostly not, mostly, always
Psychosocial demands	Are you sometimes so stressed that you do not have time to talk about or even think about something besides work?	Not at all, occasionally, roughly ¼ of the time, half the time, roughly ¾ of the time, almost all the time
	Do you sometimes have so much to do that you have to work during lunch, work overtime, or take work home?	Not at all, a few days per month, one day per week, a few days per week, every day
	Does your work require all of your attention and concentration?	Not at all, occasionally, roughly ¼ of the time, half the time, roughly ¾ of the time, almost all the time

*Job strain* is defined as the combination of job control and job demands split at their medians and was initially categorized into four categories: high strain jobs (low control/high demands); low strain jobs (high control/low demands); passive jobs (low control/low demands); and active jobs (high control/high demands). To make our results more comparable to some of the more influential studies on job strain (Kivimäki et al., 2012; Madsen et al., 2017), we further dichotomized this variable in order to compare high strain jobs to all other categories.

#### Outcome

*Diagnosis of depression* is taken from both in-patient and out-patient registers. The in-patient register includes hospitalizations while the outpatient register includes specialist psychiatric care. Visits to primary care are not included in these registers. Cases of depression are defined as those with any diagnosis of depression whether primary or contributing during the follow-up period between 2006 and 2016 using the ICD 10 definition of depression diagnosis (F32 and F33).

#### Covariates

*Highest obtained education* was reported in the LISA register from the year 2005. From seven original categories, we categorized this variable as (1) primary and lower secondary school or less (⩽9 years); (2) secondary (10–11 years); (3) upper-secondary (12 years); (4) post-secondary/university, 2 years or less (13–15 years); and (5) more than 3 years of post-secondary/university (>15 years).

Information on *birth country* and *civil status* is taken from the LISA register from 2005. The former was dichotomized to reflect whether the individual was born in Sweden or not. The latter was categorized as married/registered partner, divorced/separated partner, and widowed/surviving partner where partner refers to same-sex partners before marriage was legally recognized. *Number of children* was also taken from the LISA register in 2005 and was categorized as no children, one to two children, three to four children, and more than four children between the ages of 0 and 19. *Age* is derived from the index person's birth year. *Gender* was obtained from the individual's current personal registration number.

*Previous psychiatric diagnosis* was obtained from the in-patient register and was defined as having any psychiatric diagnosis with the ICD codes F00 to F99 before the baseline year of 2005.

*Parents' SEP during the index person's childhood* was obtained by linking the index person to their parents' census information from 1960, 1970, or 1980 when the index person was between the ages of 5 and 15. Occupational information was taken primarily from the father, but when this information was missing, the mother's occupation was used. The parents' SEP was classified as non-manual employees at a higher level, non-manual employees at an intermediate level, assistant non-manual employees, skilled manual workers, non-skilled manual workers, farmers, or those with no parental occupation reported.

*Parents' psychiatric diagnoses* were obtained by linking the index person to their parents' inpatient records from 1973 onward. This variable indicates whether either parent had a first-time psychiatric diagnosis prior to age 65.

### Statistical analysis

Baseline characteristics of the study population were explored according to gender and depression diagnosis during follow-up and according to the quintile categories of job control and demands.

Cox proportional hazard regression models with age as the underlying timescale were built for men and women separately to estimate hazard ratios and 95% confidence intervals for the associations between exposure to different levels of job control, job demands, and job strain in 2005 and diagnosis of depression during the follow-up period. Person-time was counted from 1 January 2006 until diagnosis of depression, emigration, death, or the end of the follow-up period on 31 December 2016, whichever came first.

Model 1 shows the crude associations with no adjustment for covariates, though age is accounted for as the underlying time scale. Model 2 is adjusted for birth year, birth country, civil status, number of children, previous psychiatric diagnosis, parents' SEP during childhood, and parents' previous psychiatric diagnoses. Model 3 is further adjusted for obtained education and mutually adjusted for job demands and control.

Covariates were chosen because they have been identified as important potential confounders in previous studies (Grynderup et al., 2012; Harvey et al., 2018; Samuelsson, Ropponen, Alexanderson, & Svedberg, 2013; Svane-Petersen et al., 2020) and may theoretically be related to both the occupation that a person has as well as their likelihood of receiving a depression diagnosis. The covariates in Model 2 are related to background and sociodemographic factors determined early in life, while the additional covariates in Model 3 are those likely to be directly related to the work environment.

In order to further investigate whether adjusting for previous psychiatric diagnoses provided adequate and appropriate control for confounding, we performed the same analyses after excluding those with any psychiatric diagnosis prior to baseline rather than adjusting for previous psychiatric diagnoses.

In order to examine whether associations were stronger based on the severity of the outcome, we also looked only at inpatient diagnoses of depression, which are the more severe diagnoses, rather than combining both inpatient and outpatient depression diagnoses. There may also be important gender differences in these different types of service use because women may be more likely to receive outpatient care before the depression becomes severe enough to require hospitalization.

To investigate whether the relationship between job strain factors and depression differed according to age, models were stratified according to three categorized age groups (30–39, 40–49, and 50–60).

Analyses were done using SAS Enterprise Guide 7.1 (SAS Institute, Cary, NC, USA).

## Results

Men and women who, at baseline, were younger, born outside of Sweden, unmarried or divorced, lower educated, had a previous psychiatric diagnosis, had parents without a recorded SEP, or parents with a psychiatric diagnosis were more likely to receive a depression diagnosis during the follow-up period (Table 2). Men without children at baseline were also more likely to develop depression during the follow-up period. Online Supplementary Table S1 shows the distribution of covariates according to job control and demands in their quintile categorization. Most notably, low education and pre-baseline psychiatric diagnoses were more common at lower levels of both job control and demands.

**Table 2.** Baseline covariates according to diagnosis of depression during the follow-up period for men and women

	Men				Women			
Depression diagnosis		Yes		No		Yes		No	
Baseline characteristics		*N*	%	*N*	%	*N*	%	*N*	%
Age	30–39	17 383	35	483 895	34	29 432	37	464 190	32
	40–49	17 453	35	463 999	32	27 158	34	471 307	33
	50–60	15 096	30	486 817	34	22 314	28	464 190	32
Birth country	Sweden	41 814	84	1 273 100	89	64 966	82	1 270 235	88
	Other	8098	16	161 441	11	13 923	18	177 968	12
Civil status	Married	20 313	41	721 759	50	33 780	43	782 411	54
	Unmarried	21 257	43	545 816	38	26 647	34	433 384	30
	Divorced	8084	16	160 197	11	17 329	22	211 372	15
	Widowed	278	1	6939	0	1148	1	21 145	1
Number of children	0	29 549	59	742 995	52	35 457	45	657 916	45
	1–2	16 252	33	563 304	39	34 999	44	651 314	45
	3–4	3892	8	123 400	9	8029	10	134 442	9
	>4	239	0	5012	0	419	1	4640	0
Education years^a^	>15	8327	17	280 769	20	17 371	22	351 416	24
	13–15	6810	14	211 613	15	12 396	16	243 896	17
	12	7871	16	225 167	16	12 702	16	230 357	16
	10–11	18 150	36	489 058	34	26 939	34	469 403	32
	⩽9	8774	18	228 104	16	9496	12	153 240	11
Psych diagnosis	No	38 676	77	1 379 307	96	61 034	77	1 391 159	96
	Yes	11 256	23	53 806	4	17 870	23	55 804	4
Parents' SEP^b^	Higher	2917	6	86 876	6	4610	6	84 076	6
	Intermediate	8287	17	255 339	18	12 891	16	248 961	17
	Assistant	5067	10	148 646	10	7651	10	147 194	10
	Skilled	9648	19	300 081	21	15 458	20	302 753	21
	Unskilled	13 027	26	382 002	27	20 115	25	383 678	26
	Farmer	1944	4	79 073	6	2951	4	83 765	6
	No SEP	9042	18	182 694	13	15 228	19	197 885	14
Parents' psych diagnosis	No	47 509	95	1 395 728	97	75 140	95	1 410 668	97
	Yes	2423	5	38 983	3	3764	5	37 644	3

a >15 = more than 3 years of university, 13–15 = less than 3 years of university, 12 = 3 years of upper secondary school, 10–11 = less than 3 years of upper secondary school, ⩽9 = compulsory school or less.

b Socioeconomic position: higher = non-manual employees at higher level, intermediate = non-manual employees at intermediate level, assistant = assistant non-manual employees, skilled = skilled manual workers, unskilled = unskilled manual workers, no SEP = no parental occupation reported.

Around 3% of men and 5% of women received a depression diagnosis during the follow-up period. For men around 22% of these diagnoses came from the inpatient register and for women around 19% were diagnosed in the inpatient register. The rest, 78% and 81%, respectively, came from the outpatient register.

Lower job control was associated with an increased risk of depression among men, and this showed a dose–response relationship, where decreasing job control was associated with a greater risk of receiving a depression diagnosis during the follow-up period (Table 3). Higher demands compared to the lowest category were associated with a slight decrease in the risk of depression. Having a passive or high strain job, compared to having a low strain job, was also associated with an increased risk of depression (HR 1.23, 95% CI 1.20–1.26 for passive jobs and HR 1.26, 95% CI 1.23–1.30 for high strain jobs), while having an active job was associated with a decrease in the risk of developing depression (HR 0.94). High strain jobs compared to all other jobs were associated with an increased risk of depression (HR 1.17, 95% CI 1.15–1.20).

**Table 3.** Hazard ratios and 95% confidence intervals for risk of depression diagnosis according to job control, job demands, and job strain for men

JEM	Quintiles	*N* cases (%)	Model 1	Model 2	Model 3
Job control	Low	12 594 (4)	1.76 (1.70–1.81)	1.43 (1.39–1.48)	1.43 (1.39–1.48)
	Med low	11 630 (4)	1.62 (1.57–1.67)	1.35 (1.31–1.39)	1.30 (1.26–1.35)
	Med	10 256 (3)	1.43 (1.39–1.47)	1.22 (1.19–1.26)	1.24 (1.20–1.28)
	Med high	8708 (3)	1.17 (1.13–1.21)	1.11 (1.07–1.15)	1.09 (1.06–1.13)
	High	6853 (2)	1	1	1
Job demands	Low	13 302 (4)	1	1	1
	Med low	9582 (3)	0.76 (0.74–0.78)	0.87 (0.84–0.89)	0.85 (0.83–0.87)
	Med	8806 (3)	0.67 (0.65–0.68)	0.82 (0.79–0.84)	0.88 (0.86–0.91)
	Med high	9398 (3)	0.72 (0.70–0.74)	0.86 (0.84–0.89)	0.91 (0.88–0.93)
	High	8953 (3)	0.71 (0.69–0.73)	0.88 (0.86–0.91)	0.94 (0.91–0.97)
Job strain	Passive	19 876 (4)	1.41 (1.37–1.44)	1.23 (1.20–1.27)	1.23 (1.20–1.26)
	Low strain	8220 (3)	1	1	1
	Active	11 767 (3)	0.87 (0.85–0.90)	0.94 (0.91–0.96)	0.94 (0.91–0.97)
	High strain	10 178 (4)	1.37 (1.33–1.41)	1.26 (1.22–1.30)	1.26 (1.23–1.30)
Job strain	High strain	10 178 (4)	1.23 (1.21–1.26)	1.16 (1.14–1.19)	1.17 (1.15–1.20)
	Other	39 863 (3)	1	1	1

Model 1 is adjusted for age.

Model 2 is adjusted for age, birth year, previous psychiatric diagnosis, civil status, number of children, immigrant status, parents' socioeconomic position, and parents' psychiatric diagnoses.

Model 3 is adjusted for age, birth year, previous psychiatric diagnosis, civil status, number of children, immigrant status, parents' socioeconomic position, parents' psychiatric diagnoses, obtained education, and job control and demands are mutually adjusted.

For women, lower job control was also associated with an increased risk of depression compared to the highest category, but this did not show a dose–response pattern, as the lowest job control category showed a weaker association than the medium-low category (HR 1.27, 95% CI 1.24–1.30 and HR 1.47, 95% CI 1.44–1.51, respectively) (Table 4). Higher job demands compared to the lowest job demands category tended to be associated with a slight decrease in risk for depression, except in the medium-low category. Passive jobs compared to low strain jobs were associated with a slight increase in the risk of depression (HR 1.12, 95% CI 1.10–1.14), while active jobs were associated with a slight decrease in risk (HR 0.84, 95% CI 0.82–0.86), and high strain jobs showed no association. When comparing high strain jobs to all other types of jobs for women, there was a very slight increase in the risk of depression (HR 1.04, 95% CI 1.02–1.06).

**Table 4.** Hazard ratios and 95% confidence intervals for risk of depression diagnosis according to job control, job demands, and job strain for women

JEM	Quintiles	*N* cases (%)	Model 1	Model 2	Model 3
Job control	Low	13 084 (5)	1.36 (1.33–1.39)	1.26 (1.23–1.29)	1.27 (1.24–1.30)
	Med low	24 058 (6)	1.71 (1.67–1.75)	1.46 (1.42–1.49)	1.47 (1.44–1.51)
	Med	15 207 (5)	1.41 (1.38–1.45)	1.30 (1.26–1.33)	1.28 (1.24–1.31)
	Med high	14 915 (5)	1.32 (1.29–1.35)	1.20 (1.17–1.23)	1.18 (1.15–1.21)
	High	11 760 (4)	1	1	1
Job demands	Low	16 838 (6)	1	1	1
	Med low	18 492 (6)	1.05 (1.03–1.08)	1.06 (1.04–1.08)	1.01 (0.99–1.04)
	Med	16 947 (5)	0.94 (0.92–0.96)	0.99 (0.97–1.02)	0.89 (0.87–0.92)
	Med high	13 500 (4)	0.76 (0.74–0.77)	0.86 (0.84–0.88)	0.89 (0.87–0.91)
	High	13 247 (4)	0.76 (0.74–0.78)	0.87 (0.85–0.89)	0.83 (0.81–0.86)
Job strain	Passive	29 517 (6)	1.16 (1.14–1.18)	1.12 (1.10–1.14)	1.12 (1.10–1.14)
	Low strain	17 820 (5)	1	1	1
	Active	17 544 (4)	0.76 (0.74–0.78)	0.84 (0.82–0.86)	0.84 (0.82–0.86)
	High strain	14 143 (5)	0.90 (0.88–0.92)	0.98 (0.96–1.00)	0.99 (0.96–1.01)
Job strain	High strain	14 143 (5)	0.92 (0.90–0.93)	0.99 (0.97–1.01)	1.04 (1.02–1.06)
	Other	64 881 (5)	1	1	1

Model 1 is adjusted for age.

Model 2 is adjusted for age, birth year, previous psychiatric diagnosis, civil status, number of children, immigrant status, parents' socioeconomic position, and parents' psychiatric diagnoses.

Model 3 is adjusted for age, birth year, previous psychiatric diagnosis, civil status, number of children, immigrant status, parents' socioeconomic position, parents' psychiatric diagnoses, obtained education, and job control and demands are mutually adjusted.

For both men and women, crude associations were attenuated after adjusting for birth year, previous psychiatric diagnosis, civil status, number of children, immigrant status, parents SEP during childhood, and parents' psychiatric diagnoses, and were generally very slightly further attenuated after additionally adjusting for education, and mutually adjusting for job control and demands.

When analyses were repeated after excluding those with a psychiatric diagnosis prior to baseline, estimates in the adjusted models were similar with only slight variation (online Supplementary Tables S2 and S3).

Using only inpatient diagnosis of depression rather than the combination of both inpatient and outpatient diagnoses resulted in similar associations for men and slightly stronger associations for women for the relationship between job control and depression diagnosis (online Supplementary Tables S4 and S5). The associations for higher demands showed a very slight reduction in risk for men and little change for women. The different categories of job strain were quite similar for both men and women when using only inpatient depression diagnoses compared to both inpatient and outpatient diagnoses.

Stratifying by age group showed virtually no change in the associations (data not shown).

## Discussion

In this register-based study of approximately 3 million Swedish workers, we found that for both men and women, lower job control, measured by decision authority at work, was associated with an increased risk of depression during the follow-up period, even after adjusting for pre-baseline psychiatric diagnoses, background sociodemographic factors, and other factors related to the working environment. These associations, however, only showed a dose–response relationship among men. Additionally, having high job demands was associated with a slight decrease in the risk of developing depression during the follow-up period for both men and women. High strain and passive jobs, that is, jobs with low control were associated with an increased risk of developing depression for men, and passive jobs were associated with an increased risk of developing depression for women.

Several previous studies have found a similar relationship between lower job control and a greater risk of depression or common mental disorders (Harvey et al., 2018; Samuelsson et al., 2013; Svane-Petersen et al., 2020). For example, one Danish study reported findings in the same direction when using a JEM measuring job control and registered diagnoses of depression (Svane-Petersen et al., 2020). That study, however, does not allow comparisons of dose–response patterns.

Previous studies have also found consistently different patterns between men and women in terms of the relationship between job strain factors and depression or other common mental health disorders (Cohidon, Santin, Chastang, Imbernon, & Niedhammer, 2012; Virtanen et al., 2007; Wieclaw et al., 2008). These associations tend to be weaker among women. That women in the lowest job control category had a lower risk of developing depression than women in the medium-low category is a somewhat puzzling result, though most previous studies do not allow for dose–response comparisons. The lowest job control category for both men and women tended to have a higher proportion of highly educated individuals compared to all other categories besides the highest (online Supplementary Table S1). A closer investigation into the occupations included in the different categories of job control revealed that the lowest category of job control included physicians and a large group of primary school teachers among women, that is, individuals with higher education and perhaps a more prestigious position in society. Women in the medium-low category were almost exclusively lower-level healthcare workers, while men in the equivalent group had a large range of different occupations. Thus, one possible explanation could be that women in the lowest job control category may have a lower risk of depression due to having higher status jobs that require higher education and career planning and women in the medium-low category may have an increased risk of depression due to unmeasured factors related to working in lower-level healthcare jobs. One such factor may be emotional demands (Vammen et al., 2016). However, excluding doctors and primary school teachers did not result in a different pattern of association.

Gender differences in depression are persistent and universal with depression being more common in women (Steel et al., 2014). Women may be disproportionately exposed to additional risk factors for depression that are unrelated to the characteristics of the working environment. Thus, depression cases are more evenly spread across different levels of education and different occupations among women compared to men. If these additional depressions are in fact related to something other than the psychosocial work environment, then we would indeed expect weaker associations among women. Though no single factor has been found to explain the gender difference in depression, it has been suggested that this may be partially explained by women more often being exposed to stressful life events and important differences in physiological as well as emotional reactions to stress (Nolen-Hoeksema, 2002).

That higher job demands were not associated with an increased risk of developing depression deviates from the expectation based on the theoretical model of job strain (Karasek, 1979). However, several previous studies which also use JEM or other aggregated measures of job demands show null results (Cohidon et al., 2012; Grynderup et al., 2012) or results similar to our own, suggesting a decrease in the risk of depression when job demands are higher (Samuelsson et al., 2013; Wieclaw et al., 2008). Podsakoff, LePine, and LePine (2007) also make a distinction between challenge stressors and hindrance stressors in the workplace, where the former gives an opportunity for development and the latter relates to issues such as role conflict and employment insecurity. It may be that jobs classified as having high demands in our study benefit individuals through challenges and opportunities, and thus may be related to a decreased risk of depression. Two high-quality population-based cohort studies reported opposite findings. That is, higher demands were associated with a greater risk of depression (Harvey et al., 2018; Virtanen et al., 2007). These studies, however, relied on individuals to report their own experience of job demands. The individual experience of job demands has been found to be strongly affected by reporter bias and to overestimate associations (Kolstad et al., 2011). Thus, what is captured on an individual level may reflect differences in personality, working style, and depressive disposition.

Two important meta-analyses have reported overall associations between job strain and depression which were higher than what we found in the present study (Madsen et al., 2017; Theorell et al., 2015). The summary odds ratio in a meta-analysis conducted by Theorell et al. (2015) was 1.74 (95% CI 1.53–1.96). In a meta-analysis by Madsen et al. (2017), the summary risk ratio was 1.77 (95% CI 1.47–2.13). However, when the latter included only unpublished datasets using hospital diagnosis of depression rather than diagnostic interviews, the summary risk ratio was lower (RR 1.27, 95% CI 1.04–1.55). That job control is the more important and driving force of the job strain model has been postulated in relation to coronary heart disease as well as depression (Ingre, 2015; Mikkelsen et al., 2018) and has been debated among experts (Kivimäki et al., 2015). Specifically, using quadrants or a dichotomous measure to represent job strain has been criticized as being uninformative as these associations can be driven by only one of the factors in these combined categories. Furthermore, there is evidence supporting job control alone as a more important predictor of depression and little evidence for an interaction between control and demand (Mikkelsen et al., 2018; Theorell et al., 2015). Our separate analysis of job control and job demands, as well as the more extensive four-category job strain categorization including passive jobs, also reveals that on the aggregated level, job control appears to be the more important predictor of depression.

Strengths of this study include the large sample size encompassing legally working individuals between the ages of 30 and 60 during 2005 in the Swedish population. This substantially reduces bias due to selection and attrition, which some previous studies have been criticized for (Choi et al., 2015). That the psychosocial workplace environment was measured independently of the index person using JEM measures is also a strength that provides better evidence for a causal interpretation. Additionally, the use of patient registers for defining depression diagnoses is also a more objective measure than relying on self-report. Furthermore, the patient registers allowed for the possibility to account for those with a previous psychiatric diagnosis prior to baseline. The ability to link the index person to their parents in order to obtain information on SEP during childhood and parents' psychiatric diagnoses was also a strength. Finally, that we found similar associations when only considering the more severe inpatient diagnoses of depression shows that our results were not sensitive to the type and severity of depression diagnosis.

This study is not without its limitations. First, though the use of JEM scores allows for a more independent measure of the workplace psychosocial environment, they do not consider inter-individual variation in exposure levels within particular occupations. In other words, the JEM scores serve as an aggregated experience for people within their occupations, but do not necessarily reflect the work environment that the index person experiences themselves. Second, though patient registers are rather objective measures of depression diagnosis, they only capture more severe cases including those who get hospital or specialized treatment and miss milder untreated cases or cases treated in primary care. It is important to note, however, that this Swedish healthcare system is tax-funded with limited financial barriers for care-seeking. Furthermore, there are no validation studies, to our knowledge, of depression diagnoses in the Swedish patient registers, though these registers have been found to have high coverage of psychiatric diagnoses in general (Ludvigsson et al., 2011). Further studies evaluating depression diagnoses in the Swedish patient registers are needed. Third, psychiatric diagnoses for both the index person and their parents were only available after 1973. Thus, pre-baseline psychiatric diagnoses for the index person and parents' diagnoses prior to 1973 would be missed. However, later diagnoses can arguably be used as a proxy for earlier psychiatric problems. Fourth, as with all observational studies, there is a risk for residual confounding. There may be particular differences within psychosocial workplace environments that are also differently related to depression diagnosis that we did not account for. Fifth, Sweden and the other Nordic countries have unique labor market and social welfare policies which may make it difficult to directly generalize the results to other countries or regions. Finally, using one baseline measure of job exposure, while more comparable to previous studies, does not address accumulation or change in occupational exposures over time, which was beyond the scope of the present study.

That lower job control is associated with an increased risk of developing depression is an important finding for future interventions aiming at improving job control or finding other ways to support those with low control jobs with the potential to improve mental health. A synthesis of systematic reviews found that the best evidence indicated that multi-dimensional interventions aimed at decreasing job demands and increasing job control tend to be related to less absenteeism, financial benefits, and increased productivity or performance, but concluded with the need for more research on interventions targeting job control (Williams-Whitt et al., 2015).

## Supplementary material

For supplementary material accompanying this paper visit https://doi.org/10.1017/S003329172100060X. click here to view supplementary material
