# Supplementary material for: Psychosocial working conditions and the risk of diagnosed depression: a Swedish register-based study
Source: Psychol Med. 2021 Mar 8;52(15):3730–8. doi: 10.1017/S003329172100060X (PMC9772906; doi:10.1017/S003329172100060X)
Supplement: Supplementary file 1 [file S003329172100060Xsup001.docx]

**Supplementary tables**

**Table S1** Baseline characteristics according to job control and job demands

|  | |  | | | |  |  | |  | |  |  | |  |  | | |  | |  | |  |  |
| --- | --- | --- | --- | --- | --- | --- | --- | --- | --- | --- | --- | --- | --- | --- | --- | --- | --- | --- | --- | --- | --- | --- | --- |
|  | |  | | | | **Men** | |  |  | |  |  | | **Women** | | |  |  | |  | |  |  |
| **Job control** | |  | | | | **Low** | **Med low** | | **Med** | | **Med high** | **High** | | **Low** | **Med low** | | | **Med** | | **Med high** | | **high** |  |
|  | |  | | | | **%** | **%** | | **%** | | **%** | **%** | | **%** | **%** | | |  | | **%** | | **%** |  |
| Age | | 30-39 | | | | 36 | 34 | | 34 | | 32 | 32 | | 34 | 31 | | | 32 | | 30 | | 35 |  |
|  | | 40-49 | | | | 33 | 33 | | 32 | | 32 | 32 | | 31 | 34 | | | 33 | | 33 | | 32 |  |
|  | | 50-60 | | | | 31 | 33 | | 34 | | 35 | 35 | | 35 | 35 | | | 36 | | 37 | | 33 |  |
| Immigrant | | | |  | | 19 | 12 | | 12 | | 7 | 7 | | 14 | 15 | | | 11 | | 13 | | 9 |  |
| Civil status | | Married | | | | 45 | 45 | | 47 | | 53 | 60 | | 54 | 52 | | | 53 | | 53 | | 55 |  |
|  | | Unmarried | | | | 41 | 42 | | 41 | | 36 | 30 | | 30 | 29 | | | 31 | | 30 | | 31 |  |
|  | | Divorced | | | | 13 | 13 | | 12 | | 10 | 9 | | 14 | 17 | | | 15 | | 16 | | 13 |  |
|  | | Widowed | | | | 0 | 1 | | 0 | | 1 | 0 | | 1 | 2 | | | 1 | | 2 | | 1 |  |
| Number of | | 0 | | | | 54 | 55 | | 53 | | 50 | 47 | | 44 | 43 | | | 45 | | 48 | | 47 |  |
| children | | 1-2 | | | | 36 | 36 | | 38 | | 41 | 43 | | 45 | 45 | | | 45 | | 44 | | 46 |  |
|  | | 3-4 | | | | 9 | 8 | | 8 | | 8 | 9 | | 10 | 12 | | | 9 | | 8 | | 7 |  |
|  | | >4 | | | | 1 | 0 | | 0 | | 0 | 0 | | 0 | 1 | | | 0 | | 0 | | 0 |  |
| Education | | >15 | | | | 19 | 8 | | 10 | | 15 | 46 | | 36 | 12 | | | 19 | | 21 | | 37 |  |
| Years ^a^ | | 13-15 | | | | 11 | 12 | | 9 | | 21 | 21 | | 11 | 11 | | | 27 | | 16 | | 20 |  |
|  | | 12 | | | | 15 | 16 | | 15 | | 19 | 14 | | 11 | 18 | | | 14 | | 19 | | 17 |  |
|  | | 10-11 | | | | 37 | 42 | | 46 | | 31 | 14 | | 28 | 50 | | | 29 | | 30 | | 20 |  |
|  | | <9 | | | | 19 | 22 | | 19 | | 14 | 6 | | 14 | 10 | | | 11 | | 14 | | 6 |  |
| Demands | | High | | | | 26 | 8 | | 6 | | 15 | 45 | | 49 | 14 | | | 12 | | 9 | | 22 |  |
|  | | Med high | | | | 16 | 20 | | 11 | | 32 | 22 | | 10 | 0 | | | 33 | | 28 | | 32 |  |
|  | | Med | | | | 12 | 15 | | 12 | | 33 | 28 | | 1 | 56 | | | 9 | | 12 | | 13 |  |
|  | | Med low | | | | 27 | 12 | | 51 | | 3 | 4 | | 29 | 28 | | | 7 | | 6 | | 29 |  |
|  | | Low | | | | 19 | 45 | | 20 | | 18 | 0 | | 12 | 1 | | | 39 | | 45 | | 4 |  |
| Psych diag | |  | | | | 5 | 6 | | 5 | | 4 | 2 | | 4 | 6 | | | 5 | | 5 | | 3 |  |
| Parents’ SEP ^b^ | | Higher | | | | 5 | 4 | | 3 | | 6 | 12 | | 6 | 3 | | | 5 | | 6 | | 10 |  |
|  | | Intermediate | | | | 14 | 13 | | 14 | | 21 | 27 | | 16 | 12 | | | 17 | | 17 | | 24 |  |
|  | | Assistant | | | | 9 | 9 | | 9 | | 12 | 13 | | 10 | 8 | | | 11 | | 11 | | 12 |  |
|  | | Skilled | | | | 20 | 22 | | 25 | | 21 | 16 | | 20 | 23 | | | 22 | | 21 | | 18 |  |
|  | | Unskilled | | | | 29 | 32 | | 29 | | 25 | 18 | | 26 | 31 | | | 27 | | 26 | | 21 |  |
|  | | Farmer | | | | 4 | 6 | | 5 | | 7 | 5 | | 6 | 6 | | | 6 | | 5 | | 5 |  |
|  | | No SEP | | | | 20 | 14 | | 13 | | 9 | 9 | | 16 | 17 | | | 13 | | 14 | | 10 |  |
| Parents’ psych | |  | | | | 3 | 3 | | 3 | | 3 | 2 | | 3 | 3 | | | 3 | | 3 | | 2 |  |
| **Demands** | |  | | | |  |  | |  | |  |  | |  |  | | |  | |  | |  |  |
| Age | | 30-39 | | | | 34 | 36 | | 38 | | 33 | 29 | | 32 | 33 | | | 31 | | 35 | | 30 |  |
|  | | 40-49 | | | | 34 | 33 | | 32 | | 32 | 33 | | 33 | 31 | | | 35 | | 33 | | 32 |  |
|  | | 50-60 | | | | 33 | 32 | | 31 | | 35 | 38 | | 36 | 35 | | | 34 | | 32 | | 38 |  |
| Immigrant | |  | | | | 17 | 11 | | 8 | | 9 | 11 | | 17 | 15 | | | 13 | | 9 | | 10 |  |
| Civil status | | Married | | | | 42 | 46 | | 50 | | 52 | 60 | | 50 | 51 | | | 54 | | 54 | | 59 |  |
|  | | Unmarried | | | | 44 | 43 | | 40 | | 35 | 29 | | 32 | 32 | | | 30 | | 31 | | 26 |  |
|  | | Divorced | | | | 13 | 11 | | 10 | | 12 | 11 | | 16 | 16 | | | 15 | | 14 | | 14 |  |
|  | | Widowed | | | | 1 | 0 | | 0 | | 1 | 1 | | 2 | 2 | | | 1 | | 1 | | 1 |  |
| Number of | | 0 | | | | 57 | 53 | | 51 | | 51 | 48 | | 49 | 47 | | | 43 | | 44 | | 44 |  |
| children | | 1-3 | | | | 35 | 38 | | 40 | | 40 | 43 | | 42 | 43 | | | 46 | | 48 | | 47 |  |
|  | | 3-4 | | | | 8 | 8 | | 8 | | 8 | 10 | | 9 | 10 | | | 10 | | 8 | | 9 |  |
|  | | >4 | | | | 0 | 0 | | 0 | | 0 | 0 | | 0 | 0 | | | 0 | | 0 | | 0 |  |
| Education | | >15 | | | | 4 | 9 | | 21 | | 22 | 43 | | 7 | 9 | | | 8 | | 34 | | 62 |  |
| Years ^a^ | | 13-15 | | | | 7 | 8 | | 24 | | 18 | 16 | | 9 | 11 | | | 12 | | 31 | | 21 |  |
|  | | 12 | | | | 16 | 16 | | 17 | | 16 | 14 | | 18 | 19 | | | 22 | | 13 | | 6 |  |
|  | | 10-11 | | | | 48 | 47 | | 29 | | 29 | 19 | | 43 | 43 | | | 51 | | 17 | | 9 |  |
|  | | <9 | | | | 26 | 20 | | 10 | | 15 | 9 | | 23 | 17 | | | 7 | | 5 | | 2 |  |
| Psych diag | |  | | | | 7 | 5 | | 4 | | 4 | 3 | | 6 | 6 | | | 5 | | 4 | | 4 |  |
| Parents’ SEP ^b^ | | Higher | | | | 2 | 3 | | 7 | | 7 | 11 | | 3 | 4 | | | 4 | | 8 | | 10 |  |
|  | | Intermediate | | | | 11 | 14 | | 21 | | 20 | 23 | | 12 | 14 | | | 14 | | 23 | | 23 |  |
|  | | Assistant | | | | 7 | 8 | | 11 | | 12 | 13 | | 8 | 9 | | | 9 | | 12 | | 12 |  |
|  | | Skilled | | | | 23 | 26 | | 20 | | 19 | 16 | | 22 | 22 | | | 23 | | 19 | | 18 |  |
|  | | Unskilled | | | | 32 | 31 | | 24 | | 26 | 20 | | 30 | 29 | | | 30 | | 23 | | 20 |  |
|  | | Farmer | | | | 5 | 6 | | 6 | | 6 | 4 | | 5 | 6 | | | 6 | | 5 | | 6 |  |
|  | | No SEP | | | | 19 | 12 | | 10 | | 11 | 12 | | 19 | 16 | | | 14 | | 10 | | 11 |  |
| Parents’ psych | |  | | | | 3 | 3 | | 3 | | 3 | 2 | | 3 | 3 | | | 3 | | 3 | | 2 |  |
|  |  |  |  | |  | |  | | |  | |  |  | | |  | | |  | |  | | |

*^a^ >15 = more than 3 years of university, 13-15 = less than 3 years of university, 12 = 3 years of upper secondary school, 10-11 = less than 3 years of upper secondary school, <9 = compulsory school or less.*

*^b^ Socioeconomic position: higher = non-manual employees at higher level, intermediate = non-manual employees at intermediate level, assistant = assistant non-manual employees, skilled = skilled manual workers, non-skilled manual workers, no SEP = no parental occupation reported.*

**Table S2** Hazard ratios and 95% confidence intervals for risk of depression diagnosis according to job control, job demands, and job strain for men excluding those with any psychiatric diagnosis prior to baseline

| **JEM** | **Quintiles** | **N cases (%)** | **Model 1** | **Model 2** | **Model 3** |
| --- | --- | --- | --- | --- | --- |
| Job | Low | 9,639 (3) | 1.65 (1.59-1.70) | 1.50 (1.45-1.55) | 1.46 (1.41-1.51) |
| control | Med low | 8,643 (3) | 1.48 (1.43-1.53) | 1.41 (1.36-1.46) | 1.31 (1.26-1.36) |
|  | Med | 7,843 (3) | 1.34 (1.29-1.38) | 1.28 (1.24-1.33) | 1.27 (1.22-1.32) |
|  | Med high | 6,900 (2) | 1.12 (1.08-1.16) | 1.12 (1.08-1.16) | 1.08 (1.04-1.12) |
|  | High | 5,744 (2) | 1 | 1 | 1 |
| Job | Low | 9,632 (3) | 1 | 1 | 1 |
| demands | Med low | 7,396 (3) | 0.79 (0.77-0.82) | 0.84 (0.82-0.87) | 0.83 (0.80-0.85) |
|  | Med | 7,024 (2) | 0.71 (0.69-0.73) | 0.77 (0.75-0.80) | 0.86 (0.83-0.89) |
|  | Med high | 7,441 (3) | 0.77 (0.74-0.79) | 0.82 (0.80-0.85) | 0.89 (0.86-0.92) |
|  | High | 7,276 (3) | 0.77 (0.75-0.80) | 0.83 (0.80-0.85) | 0.92 (0.88-0.95) |
| Job strain | Passive | 14,893 (3) | 1.37 (1.33-1.41) | 1.28 (1.24-1.31) | 1.26 (1.22-1.30) |
|  | Low strain | 6,399 (2) | 1 | 1 | 1 |
|  | Active | 9,724 (2) | 0.92 (0.89-0.95) | 0.92 (0.89-0.95) | 0.93 (0.90-0.96) |
|  | High strain | 7,843 (3) | 1.37 (1.32-1.42) | 1.28 (1.24-1.33) | 1.29 (1.24-1.33) |
| Job strain | High strain | 7,843 (3) | 1.23 (1.20-1.26) | 1.18 (1.15-1.21) | 1.19 (1.16-1.22) |
|  | Other | 30,926 (3) | 1 | 1 | 1 |

*Model 1 is adjusted for age.*

*Model 2 is adjusted for age, birth year, civil status, number of children, immigrant status, parents’ socioeconomic position, and parents’ psychiatric diagnoses.*

*Model 3 is adjusted for age, birth year, civil status, number of children, immigrant status, parents’ socioeconomic position, parents’ psychiatric diagnoses, obtained education, and job control and demands are mutually adjusted.*

**Table S3** Hazard ratios and 95% confidence intervals for risk of depression diagnosis according to job control, job demands, and job strain for women excluding those with a psychiatric diagnosis prior to baseline

| **JEM** | **Quintiles** | **N cases (%)** | **Model 1** | **Model 2** | **Model 3** |
| --- | --- | --- | --- | --- | --- |
| Job | Low | 10,303 (4) | 1.31 (1.27-1.34) | 1.27 (1.23-1.30) | 1.27 (1.24-1.31) |
| control | Med low | 17,753 (5) | 1.56 (1.53-1.60) | 1.49 (1.45-1.53) | 1.52 (1.47-1.56) |
|  | Med | 11,791 (4) | 1.34 (1.31-1.38) | 1.32 (1.29-1.36) | 1.30 (1.26-1.34) |
|  | Med high | 11,530 (4) | 1.25 (1.22-1.29) | 1.20 (1.17-1.24) | 1.17 (1.14-1.21) |
|  | High | 9,757 (3) | 1 | 1 | 1 |
| Job | Low | 12,843 (5) | 1 | 1 | 1 |
| demands | Med low | 13,979 (5) | 1.05 (1.02-1.07) | 1.06 (1.04-1.09) | 1.02 (0.99-1.05) |
|  | Med | 12,888 (4) | 0.93 (0.91-0.95) | 0.97 (0.95-0.99) | 0.86 (0.84-0.89) |
|  | Med high | 10,860 (4) | 0.79 (0.77-0.81) | 0.84 (0.82-0.87) | 0.88 (0.86-0.91) |
|  | High | 10,564 (4) | 0.78 (0.76-0.80) | 0.84 (0.82-0.86) | 0.82 (0.79-0.84) |
| Job strain | Passive | 22,145 (5) | 1.14 (1.12-1.17) | 1.14 (1.11-1.16) | 1.14 (1.11-1.16) |
|  | Low strain | 13,656 (4) | 1 | 1 | 1 |
|  | Active | 14,192 (3) | 0.79 (0.77-0.81) | 0.82 (0.81-0.84) | 0.84 (0.82-0.86) |
|  | High strain | 11,141 (4) | 0.91 (0.89-0.93) | 0.96 (0.94-0.99) | 0.97 (0.95-1.00) |
| Job strain | High strain | 11,141 (4) | 0.93 (0.91-0.95) | 0.97 (0.95-0.99) | 1.03 (1.01-1.05) |
|  | Other | 49,993 (4) | 1 | 1 | 1 |

*Model 1 is adjusted for age.*

*Model 2 is adjusted for age, birth year, civil status, number of children, immigrant status, parents’ socioeconomic position, and parents’ psychiatric diagnoses.*

*Model 3 is adjusted for age, birth year, civil status, number of children, immigrant status, parents’ socioeconomic position, parents’ psychiatric diagnoses, obtained education, and job control and demands are mutually adjusted.*

**Table S4** Hazard ratios and 95% confidence intervals for risk of inpatient depression diagnosis according to job control, job demands, and job strain for men

| **JEM** | **Quintiles** | **N cases (%)** | **Model 1** | **Model 2** | **Model 3** |
| --- | --- | --- | --- | --- | --- |
| Job | Low | 4,463 (2) | 1.98 (1.88-2.09) | 1.54 (1.46-1.62) | 1.48 (1.40-1.56) |
| control | Med low | 4,294 (1) | 1.89 (1.80-1.99) | 1.46 (1.38-1.54) | 1.33 (1.25-1.41) |
|  | Med | 3,641 (1) | 1.60 (1.52-1.69) | 1.28 (1.21-1.35) | 1.23 (1.16-1.31) |
|  | Med high | 3,002 (1) | 1.26 (1.20-1.34) | 1.15 (1.08-1.21) | 1.09 (1.03-1.15) |
|  | High | 2,188 (1) | 1 | 1 | 1 |
| Job | Low | 5,004 (2) | 1 | 1 | 1 |
| demands | Med low | 3,408 (1) | 0.72 (0.69-0.75) | 0.84 (0.80-0.87) | 0.82 (0.79-0.86) |
|  | Med | 2,904 (1) | 0.59 (0.57-0.62) | 0.75 (0.72-0.79) | 0.83 (0.79-0.88) |
|  | Med high | 3,331 (1) | 0.67 (0.64-0.70) | 0.84 (0.80-0.88) | 0.90 (0.86-0.94) |
|  | High | 2,941 (1) | 0.61 (0.58-0.63) | 0.81 (0.78-0.85) | 0.90 (0.85-0.95) |
| Job strain | Passive | 7,314 (2) | 1.51 (1.45-1.58) | 1.31 (1.25-1.37) | 1.29 (1.23-1.35) |
|  | Low strain | 2,814 (1) | 1 | 1 | 1 |
|  | Active | 3,925 (1) | 0.83 (0.79-0.87) | 0.93 (0.89-0.98) | 0.95 (0.91-1.00) |
|  | High strain | 3,535 (1) | 1.36 (1.30-1.43) | 1.27 (1.21-1.34) | 1.28 (1.22-1.35) |
| Job strain | High strain | 3,535 (1) | 1.20 (1.16-1.25) | 1.14 (1.10-1.18) | 1.15 (1.11-1.20) |
|  | Other | 14,053 (1) | 1 | 1 | 1 |

*Model 1 is adjusted for age.*

*Model 2 is adjusted for age, birth year, previous psychiatric diagnosis, civil status, number of children, immigrant status, parents’ socioeconomic position, and parents’ psychiatric diagnosis.*

*Model 3 is adjusted for age, birth year, previous psychiatric diagnosis, civil status, number of children, immigrant status, parents’ socioeconomic position, parents’ psychiatric diagnoses, obtained education, and job control and demands are mutually adjusted.*

**Table S5** Hazard ratios and 95% confidence intervals for risk of inpatient depression diagnosis according to job control, job demands, and job strain for women

| **JEM** | **Quintiles** | **N cases (%)** | **Model 1** | **Model 2** | **Model 3** |
| --- | --- | --- | --- | --- | --- |
| Job | Low | 4,069 (2) | 1.54 (1.47-1.61) | 1.39 (1.33-1.46) | 1.37 (1.31-1.44) |
| control | Med low | 8,193 (2) | 2.10 (2.01-2.18) | 1.69 (1.62-1.76) | 1.65 (1.57-1.72) |
|  | Med | 4,774 (2) | 1.60 (1.53-1.67) | 1.41 (1.35-1.48) | 1.37 (1.31-1.44) |
|  | Med high | 4,571 (2) | 1.45 (1.39-1.52) | 1.28 (1.23-1.34) | 1.23 (1.17-1.30) |
|  | High | 3,204 (1) | 1 | 1 | 1 |
| Job | Low | 5,442 (2) | 1 | 1 | 1 |
| demands | Med low | 6,111 (2) | 1.08 (1.04-1.12) | 1.08 (1.04-1.12) | 1.02 (0.97-1.06) |
|  | Med | 5,564 (2) | 0.96 (0.92-1.00) | 1.02 (0.98-1.06) | 0.89 (0.85-0.93) |
|  | Med high | 3,791 (1) | 0.67 (0.65-0.70) | 0.78 (0.75-0.82) | 0.85 (0.82-0.89) |
|  | High | 3,903 (1) | 0.69 (0.66-0.72) | 0.82 (0.79-0.86) | 0.82 (0.78-0.87) |
| Job strain | Passive | 9,941 (2) | 1.25 (1.21-1.29) | 1.18 (1.14-1.22) | 1.17 (1.13-1.21) |
|  | Low strain | 5,681 (2) | 1 | 1 | 1 |
|  | Active | 4,993 (1) | 0.69 (0.67-0.72) | 0.78 (0.75-0.81) | 0.81 (0.78-0.85) |
|  | High strain | 4,196 (1) | 0.85 (0.81-0.88) | 0.94 (0.90-0.98) | 0.99 (0.95-1.04) |
| Job strain | High strain | 4,196 (1) | 0.86 (0.83-0.89) | 0.94 (0.91-0.97) | 1.04 (1.00-1.08) |
|  | Other | 20,615 (2) | 1 | 1 | 1 |

*Model 1 is adjusted for age.*

*Model 2 is adjusted for age, birth year, previous psychiatric diagnosis, civil status, number of children immigrant status, parents’ socioeconomic position, and parents’ psychiatric diagnoses.*

*Model 3 is adjusted for age, birth year, previous psychiatric diagnosis, civil status, number of children, immigrant status, parents’ socioeconomic position, parents’ psychiatric diagnoses, obtained education, and job control and demands are mutually adjusted.*
